# What orthopaedic surgery residents need to know about the hand and wrist?

**DOI:** 10.1186/1472-6920-7-33

**Published:** 2007-10-05

**Authors:** Veronica MR Wadey, Amy Ladd, Parvati Dev, Decker Walker

**Affiliations:** 1 Orthopaedic Surgeon, Assistant Professor, Department of Surgery, Division of Orthopaedic Surgery, The Faculty of Medicine, University of Toronto, 43 Wellesley Street East, Suite 315, Toronto ON M4Y 1H1, Canada; 2 Department of Orthopaedic Surgery, Stanford University, Stanford, USA; 3 Department of Medicine, Stanford University Medical Media and Technologies, Stanford University, Stanford, USA; 4 School of Education, Stanford University, Stanford, USA

## Abstract

**Background:**

To develop a Core Curriculum for Orthopaedic Surgery; and to conduct a national survey to assess the importance of curriculum items as judged by orthopaedic surgeons with primary affiliation non-academic. Attention for this manuscript was focused on determining the importance of topics pertaining to adult hand and wrist reconstruction.

**Methods:**

A 281-item questionnaire was developed and consisted of three sections: 1) Validated Musculoskeletal Core Curriculum; 2) Royal College of Physician and Surgeons of Canada (RCPSC) Specialty Objectives and; 3) A procedure list. A random group of 131 [out of 156] orthopaedic surgeons completed the questionnaire. Data were analyzed descriptively and quantitatively using histograms, a Modified Hotel ling's T^2^-statistic [1] with p-value determined by a permutation test, and the Benjamini-Hochberg/Yekutieli procedure

**Results:**

131/156 (84%) orthopaedic surgeons participated in this study. 27/32 items received an average mean score of at least 3.0/4.0 by all respondents thus suggesting that 84% of the items are either "probably important" or "important" to know by the end of residency (SD range 0.007–0.228). The Benjamini-Hochberg procedure demonstrated that for 80% of the 32 × 31/2 = 496 possible pairs of hand and wrist questions did not appear to demonstrate the same distribution of ratings given that one question was different from that of another question.

**Conclusion:**

This study demonstrates with reliable statistical evidence, agreement on the importance of 27/32 items pertaining to hand and wrist reconstruction is included in a Core Curriculum for Orthopaedic Surgery. Residency training programs need ensure that educational opportunities focusing on the ability to perform with proficiency procedures pertaining to the hand and wrist is taught and evaluated in their respective programs.

## Background

This paper is one section of a larger study regarding the development and validation of a core curriculum for orthopaedic surgery. One entire core curriculum was validated and ten individual analyses were completed so as to clarify the content that residents should learn during residency in orthopaedic surgery. The context of this paper pertains specifically to core curriculum items relating to hand and wrist reconstruction.

The design and development of a curriculum targeted to meet the needs of a learner is essential to any educational program [2-9]. In addition, learning to use an evidence-based approach is recommended and encouraged when making clinical decisions that affect patient care and safety [10]. The same principle would apply to establishing a curriculum. It would be important to determine what content to include and support this with evidence [11].

Musculoskeletal (MSK) conditions are recognized to be such a tremendous burden on individuals and societies that the World Health Organization has declared the years 2000 to 2010 to be the Bone and Joint Decade [12]. The main goal of the decade is to improve the quality of life for all people with MSK disorders worldwide. One aim of the decade is to improve education for health care providers at all levels. A Bone and Joint Decade Undergraduate Curriculum Group (BJDUCG) established CORE curriculum recommendations for MSK conditions targeted for undergraduate medical school education [13]. This curriculum was validated in Canada [14] among six disciplines that manage individuals with musculoskeletal conditions and contributed towards the development of one orthopaedic curriculum.

Educating orthopaedic surgeons in Canada occurs in 16 accredited academic institutions under the auspices of the Royal College of Physicians and Surgeons of Canada and the Specialty Committee for Orthopaedic Surgery. Ten educational domains may be included in the design of a Core Curriculum for Orthopaedic Surgery and may include: general content that every otheropaedic surgeon should know and understand, trauma, pediatrics and adult reconstruction of the: hip/knee, foot/ankle, shoulder/elbow, hand/wrist, spine, sports medicine and tumor conditions.

The goal of each residency-training program in Canada is to produce an orthopaedic surgeon who is able to immediately step out into the community and establish a practice. The goal of the Board of Examiners is to ensure that they are effectively and accurately evaluating candidates to fulfill the role of a safe and competent orthopaedic surgeon to effectively and safely deliver these health care services to Canadians.

Upon request of the Orthopaedic Surgery Specialty Committee for The Royal College and Physicians and Surgeons of Canada and the Chief Examiners of The Board of Examiners, a national survey to assess the importance of curriculum items determined by orthopaedic surgeons whose primary affiliation is non-academic was conducted. The reasons for this are two fold. First, the attrition rate into a full-time academic practice to any of the 16 accredited academic orthopaedic programs is low and most graduates from residency programs will assume practices at locations whose primary affiliation is non-academic. Surgeons who work in these practice settings are uniquely positioned to provide input into a curriculum that is targeted to meet the needs of community orthopaedic surgeons. Second, orthopaedic surgeons in academic centers in general are typically very sub-specialized and may not reflect what is required to practice out in the community hospitals. For the reasons mentioned a nation-wide randomization and survey of orthopaedic surgeons whose primary affiliation was non-university was conducted.

The purpose of this study was to determine the importance of core content that should be included in a Core Curriculum for Orthopaedic Surgery with a specific focus on topics that pertain specifically to the hand and wrist conditions.

The null hypothesis tested was that all items in the core curriculum for orthopaedic surgery pertaining to hand and wrist conditions are equally as important for a resident to demonstrate knowledge or perform with proficiency during residency.

## Methods

### Development of the outcome (Questionnaire)

A 281-item outcome (questionnaire) was developed. It has three sections. The first section is the previously validated international core curriculum for musculoskeletal health [14]. This study was conducted on the campuses of the 16 accredited academic institutions within Canada. A cross-sectional survey of program directors and selected educators representing six disciplines and 77 accredited academic training programs that manage patients with MSK conditions was completed. These disciplines included: Family Medicine, Sports Medicine and four specialty programs of the Royal College of Physicians and surgeons of Canada (RCPSC): Emergency Medicine, Physical Medicine and Rehabilitation, Rheumatology and Orthopaedic Surgery. The survey consisted of 80 questions covering 14 different categories relating to MSK conditions. A broad variety of topics ranging from trauma to chronic conditions in both the adult and paediatric populations were incorporated into the questionnaire. The product of this study is a validated Canadian MSK Core Curriculum (available from corresponding author upon request) (4).

Specialty objectives of the Royal College of Physicians and Surgeons of Canada specifically pertaining to orthopaedic surgery made up the second cluster of items. The third section included a complete procedure list based on procedure codebooks from across Canada. The outcome was created and compared to the curricula of the various academic institutions to ensure inclusion of important topics.

This outcome underwent a full content review by ten orthopaedic surgery reseacher/educators (FRCSC) representing adult and pediatric orthopaedic surgery from within Canada and the USA who represented both genders. The 10 individuals consisted of the two Chief Examiners representing the two Boards of Examiners, Program Directors representing The RCPSC Specialty Committee for Orthopaedic Surgery and educational leaders within the Canadian orthopaedic community. A modified outcome was developed based on the feedback of this content review and the final outcome consisted of 281 items. In this particular paper the focus was on items pertaining to hand and wrist conditions. Thirty-two items pertaining to hand and wrist topics were identified from the list of 281 items. This list of 32 items underwent a content review by an orthopaedic educator who specialized in upper-extremity. Items pertaining to emerging knowledge, foundations in basic science that would pertain to the hand and wrist section are outlined in another section of the curriculum that focuses on applied basic science topics and current concepts that would be suitable for general knowledge in orthopaedic surgery. This questionnaire is available upon request from the corresponding author. The outcome was translated into French for the purposes of data collection with the Francophone orthopedists.

### Sample question

**Figure 1:**
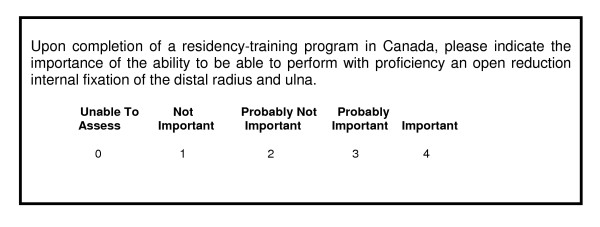
Sample question.

Each of the 281 question items were structured in a fashion similar to the sample question below that pertains to a resident's ability to perform with proficiency an open reduction and internal fixation (ORIF) of the distal radius and ulna (Figure 1). The choices for response for each question were 0 – unable to assess, 1-not important, 2-probably not important, 3-probably important and 4-important.

### Randomization & cross-sectional survey

One hundred and fifty-six orthopaedic surgeons whose primary affiliation was non-academic were randomized via a random number table to this study based on the 2004 active RCPSC list of practicing orthopaedic surgeons in Canada. The randomization was done in three separate processes to ensure appropriate representation from the Atlantic/Quebec provinces, central Canada (Ontario) and western Canada (Manitoba, Saskatchewan, Alberta and British Columbia and three territories). These distributions were based on the number of orthopaedic surgeons within these given regions.

An interview schedule was arranged and conducted in both official languages depending on the functional language of each respondent. A cross-sectional survey was completed. The Anglophone interviews were completed either over the telephone or in person and the Francophone interviews were completed via direct one-on-one interviews during a research tour through the province of Quebec.

### Statistical analysis

**Figure 2:**
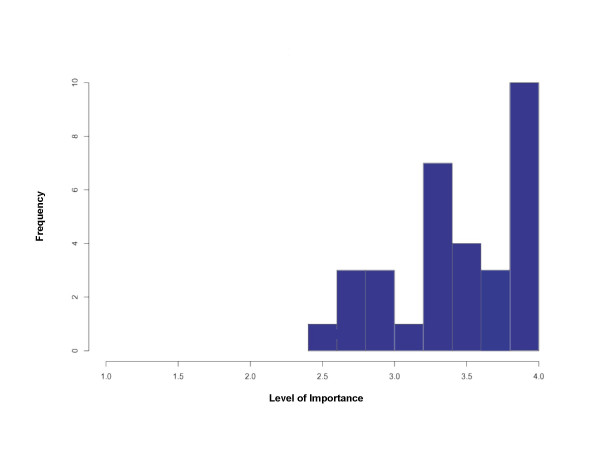
Average mean scores of hand and wrist items (N = 32).

The data was analyzed descriptively and quantitatively using histograms, the modified Hotelling's T^2^-statistic [15] with the p-value determined by a permutation test, and the Benjamini-Hochberg/Yekutieli procedure [16-18]. Our analyses assumed that each respondent answered questions independently of the answers of any other respondent, but that the answers to different questions by the same respondent might be dependent. The histogram summarizes the distribution of items by average mean scores of content pertaining specifically to the hand and wrist reconstruction (Figure 2).

One hundred and thirty-one orthopaedic surgeons answered each of the 281 questions during the same sitting. Each respondent answered each of the 281 questions. Answers given to different questions by the same respondent must be considered related ("dependant") to each other. In order to deal with these dependencies we used the modified Hotelling's T^2^-statistic with a p-value determined by a permutation test. The Benjamini-Hochberg procedure was then used to help us interpret the large number of tests we did for differences between pairs of questions. The Benjamini-Hochberg procedure showed definitively that questions are not all the same in that the distribution of the ratings given to one question is different from that of another question for many pairs of questions.

This particular study specifically studied the content pertaining to hand and wrist reconstruction. The histogram allowed us to visualize the distribution of results according to the average mean scores of each item in the curriculum pertaining to content specific to hand and wrist conditions (Figure 2).

## Results

### Demographics

A total of 131 out of 156 orthopaedic surgeons whose primary affiliation is non-academic participated in this study for an overall response rate of 84%. There was a 90% percent response rate from the Atlantic and Quebec Provinces, 80% from Ontario and the Territories and 80% from the western provinces of Manitoba, Saskatchewan, Alberta and British Columbia.

Eighty-five percent of the respondents classified themselves as generalists meaning their practice was classified as a general orthopaedic practice where they did not discriminate their patients. They diagnosed and managed a general orthopaedic practice regardless of the fellowship training they received. Most of these surgeons indicated that they completed fellowship training in areas to augment their skill sets to develop into better generalists. Fifteen percent of the respondents considered themselves to be sub-specialists, thus managing specific orthopaedic conditions with a specific area of expertise

The average age of each respondent was 48.7 years. Each respondent was in practice for an average of 16.8 years. Males made up 90% of the respondents and females 10%. The demographics of Fellowship Training Experiences are outlined in Table 1.

**Table 1:** Fellowship training experiences of respondents (N = 131)

46	**None**
23	**Combined fellowship training – Total joint reconstruction with**:
	Trauma
	Sports medicine
	Spine
	Paediatric orthopaedics
	Oncology
	Upper extremity
	Rehabilitative or general orthopaedic surgery
2	**Combined fellowship training – Paediatrics with:**
	Trauma
	Rehabilitative orthopaedics
1	**Combined fellowship training – Spine and Hand**
10	**Total joint arthroplasty (hips/knees)**
9	**Sports medicine**
8	**Spine**
7	**Hand/wrist**
6	**General orthopaedics**
5	**Upper extremity**
4	**Pediatrics**
4	**Hand/microvascular**
4	**Trauma**
2	**Foot/ankle**

### Hand and wrist reconstruction – core content

Table 2 outlines the core curriculum content pertinent to hand and wrist conditions that residents should know by the completion residency. The top and middle cluster of items consisted of both bony and soft-tissue procedures. The least important items appeared to involve more complex procedures of the hand and wrist that may require sub-specialized fellowship training prior to becoming proficient in performing these procedures.

**Table 2:** Distribution of hand and wrist conditions in ascending order of importance

**Question Number**	**Average Rank**	**SD Average Rank**	**Procedure/Content**	**Topic**
	**2.4 to 2.8**			**Probably NOT IMPORTANT Items to learn how to do with proficiency during a residency training program**
194	2.43	0.07	Procedure	Arthroplasty of the joints in the hand or fingers
177	2.69	0.06	Procedure	Diagnostic arthroscopy of the wrist
159	2.73	0.07	Procedure	Reconstruction of the collateral ligaments and/or volar plate of the metacarpal-phalangeal (MP) or interphalangeal(IP) joints of the hand
172	2.76	0.07	Procedure	Carpalmetacarpal (CMC) reconstruction
169	2.84	0.08	Procedure	Radical fasciectomy for Dupuytren's contractures
	**3.0 to 3.5**			**Items that are PROBABLY IMPORTANT ("Nice to Know") and perform with proficiency during a residency training program**
141	2.98	0.08	Procedure	Synovectomy of the hand or fingers
214	2.98	0.08	Procedure	Arthrodesis of the intercarpal bone (Intercarpal Fusion)
170	3.06	0.07	Procedure	Tendon repair about the hand or wrist
158	3.2	0.07	Procedure	Open repair of the dislocated MP/IP joints of the hand
232	3.22	0.09	Procedure	Amputation and/or disarticulation through the hand
173	3.27	0.06	Procedure	Tenodesis or tenolysis of common tendons
140	3.3	0.06	Procedure	Synovectomy of the wrist
233	3.34	0.08	Procedure	Amputation and/or disarticulation through the MP joints
213	3.37	0.06	Procedure	Arthrodesis of the wrist (total or partial meaning radius and not the ulna)
231	3.37	0.08	Procedure	Amputation and/or disarticulation through the thumb
174	3.4	0.06	Procedure	Tenotomy of common tendons
234	3.4	0.07	Procedure	Amputation and/or disarticulation through the wrist
228	3.5	0.06	Procedure	Osteotomy in the adult population through the radial and ulnar side
	**3.6 to 3.7**			**Items that are IMPORTANT ("should know") and be able to perform with proficiency during a residency training program**
136	3.6	0.06	Procedure	Arthrotomy of the hand or fingers
227	3.63	0.05	Procedure	Osteotomy of the distal radius and ulna
130	3.7	0.06	Procedure	Open reduction and internal fixation of the metacarpal bones
129	3.71	0.05	Procedure	Open reduction and internal fixation of the carpal bones (i.e.: scaphoid bone)
	**3.8 to 4.0**			**Items that are IMPORTANT ("MUST know") and be able to perform with proficiency during a residency training program**
160	3.81	0.04	Procedure	Carpal tunnel release at the wrist
249	3.82	0.04	Procedure	Bone biopsy – superficial or deep
248	3.82	0.05	Procedure	Sequestrectomy and bone grafting
135	3.9	0.03	Procedure	Arthrotomy of the wrist
166	3.91	0.23	Procedure	Open repair of the distal radius and ulnar joint (DRUJ)
54	3.96	0.02	Content	Specify the signs and symptoms, outline the assessment and investigations, propose a differential diagnosis, outline the principles of management of a patient with a primary bone and/or soft-tissue tumor
128	3.98	0.02	Content	Fasciotomy for compartment syndrome of the upper extremity
147	3.98	0.02	Content	Open reduction and internal fixation of the distal radius and ulna in the adult population
250	4	0.01	Content	Bone graft harvesting from the iliac crest, distal radius and proximal tibia
263	4	0.01	Content	Manage a closed reduction of the distal radius and ulna in the pediatric population

Twenty-seven out of thirty-two (84%) (Figure 2) pertaining to hand and wrist content and procedures, received an average mean score of ≥ 3.0/4.0 by all 131 respondents thus suggesting that 84% of the items are either probably important or important to know by the end of residency. The SD for each item ranged between 0.007 and 0.228 (Table 2). In addition, the Benjamini-Hochberg procedure demonstrated that for 80% of the 32 × 31/2 = 496 possible pairs of questions pertaining to hand and wrist did not appear to demonstrate the same distribution of ratings given that one question was different from that of another question for many pairs of questions.

The strengths of the study included: 1) a previously validated international core curriculum for musculoskeletal health 2) randomization of orthopaedic surgeons in three different regions of Canada 3) A full review of content prior to conducting the study; 4) direct one-on-one interviews that may explain the 84% response rate; 5) the use of a translated outcome and direct interviews with the Francophone orthopaedists that may explain the 90% response rate from the Quebec/Atlantic provinces and; 6) full endorsement of The Royal College of Physicians and Surgeons of Canada; the 16 academic accredited orthopaedic surgery training programs; the Canadian Orthopaedic Association and; Bone & Joint Decade Canada demonstrating an unprecedented collaboration for the sole purpose of improving education of orthopaedic surgeons across Canada.

The limitations of the study include: 1) a positive response bias existed within the questionnaire; 2) limited scale grading the level of importance and; 3) in the present study the wording of the questions asked each respondent to indicate the importance of either content or procedures. This implies that we are asking opinions rather than what they are actually doing.

## Discussion

Previous attempts have been made to define curriculum in the area of the hand and wrist [19-21]. There appears to be substantial agreement among all the orthopaedic surgeons whose primary affiliation is non-university regarding content to be included in a core curriculum for orthopaedic surgery regarding hand and wrist conditions based on the Canadian Orthopaedic Surgery Core Curriculum Recommendations outcome. This study demonstrated with reliable statistical evidence, agreement on the importance of 27/32 (84.3%) items relating to procedures of the hand and wrist, to be included in a Core Curriculum for Orthopaedic Surgery a curriculum targeted to provide orthopaedic surgeons who intend to practice as generalists. The top two clusters of items identified to be important tend to reflect common problems of the hand and wrist. Fractures to the hand are in the list of essential items to learn. Literature suggests that fractures of the hand be referred to a "hand service" [22]. If this is true then it would imply that anyone sustaining a fracture to the hand should either be referred to a tertiary care centre that has a hand service or a hand service with surgeons competent to manage these conditions needs to be available at the community hospitals. Either way, as a profession we will need to ensure that an adequate educational process is in place to meet future demands in the area of hand trauma.

Various procedures pertaining to hand and wrist reconstruction previously performed by orthopaedic surgeons have been identified as not being important to be able to do with proficiency upon completion of residency training by orthopaedists whose primary affiliation is non-academic. The least important content includes: 1) soft-tissue procedures such as radical fasciectomy for Dupuytren's contracture; carpal-metacarpal reconstruction; collateral ligament or volar plate reconstruction of the metacarpal phyalyngeal or intraphalyngeal joints; 2) arthroplasty of the joints in the hand and fingers and; 3) diagnostic arthroscopy of the wrist. The implication may be that these procedures are too complex or that residents will not or are not obtaining enough clinical exposure or hands on experience to be able to perform these procedures with proficiency by the end of residency. This may suggest that complex procedures of hand and wrist conditions require additional fellowship training.

Historically, in Canada, plastic surgeons performed surgery around the hand and wrist. However, this study demonstrates that 84% of the items in the hand and wrist section were ranked as being important for residents in orthopaedic surgery to learn during residency. This would suggest that this population of orthopaedic surgeons have the opinion that common conditions of the wrist and hand are important for orthopaedic surgeons in non-university practices to be able to perform with proficiency. Only 16 accredited academic centers exist within Canada. Graduating orthopaedic surgeons may not choose to practice in a university setting and referring to tertiary care centers is becoming more difficult. Obtaining and maintaining skill sets associated with the hand and wrist may become more essential for those surgeons wishing to practice in non-university settings. Hence, these individuals will need to be very good generalists or establish themselves in a group practice where complimentary subspecialty skill sets exist within the group of practicing surgeons.

This study captured the opinion of a particular group of surgeons whose practice pattern was non-university and where most classified themselves as general orthopaedic surgeons. The study may have been stronger if a direct correlation between the opinions of each surgeon was mapped with the number of procedures performed by a surgeon relative to the surgeons practice. Another suitable future study would focus on obtaining the opinions of the university based orthopaedic surgeons and comparing with the results of this study.

Programs may need to ensure that appropriate educational opportunity that might not have been in place during the past is now made available. It will be important for residents to have the opportunity to focus on content and the ability to perform surgical procedures associated with the hand and wrist with proficiency so that they may be adequately prepared for community practice.

Historically, the Halstedian method of "see one, do one and teach one" has been the method of choice for teaching surgery residents how to perform procedures. This method of teaching increases operating room time, use of equipment and human resources[23]. In addition, residents are dependent on surgery by serendipity. They may not have the opportunity to learn a particular surgical procedure unless a patient is scheduled for a particular procedure at a teaching hospital. Another potential problem is that residents may in fact be attending procedures in the operating room but are not given the opportunity to actually operate for reasons that are not the focus of this study. Finally, there is a lack of objective measures against which residents are evaluated [24].

Terminal and enabling objectives for orthopaedic curricula should focus on emergent and common problems in the educational domain of the hand and wrist with the fellowship examination process reflecting these objectives.

## Conclusion

The study demonstrates clusters of various topics with respect to their level of importance in a core curriculum for orthopaedic surgery. Hence, the null hypothesis stating that all Items pertaining to hand and wrist conditions are equally as important for a resident to demonstrate knowledge or perform with proficiency during residency must be rejected.

The results of this study suggest that common procedures associated with hand and wrist conditions are necessary for care orthopaedic surgeons in a non-university practice to be able to perform. This is especially the case in hand trauma. Orthopaedic Surgery Residency Programs are uniquely positioned to make suggestions, provide solutions to ensure adequate surgical training in the area of the hand and wrist meets the needs of the many orthopaedic surgeons in Canada whose primary affiliation is non-academic. If this goal is not achieved, a strategic plan facilitating the acquisition of an optimal clinical experience for each resident may need to be developed and implemented into training programs. This may be in the form of continued direct hands on experience in the operating room that may be structured differently or possibly through the development and implementation of surgical or virtual skills labs. Either way, it will be essential for the academic programs within Canada to remain unified in their approach to educating our future orthopaedic surgeons in order to combat the present and projected burden of illness resulting from musculoskeletal conditions and in particular conditions pertaining to the hand and wrist.

## Competing interests

The authors declare that they have no competing interests.

## Authors' contributions

VW was the principle investigator and was responsible for the grant proposal, data collection, and interpretation of the analyses and writing of the manuscript. AL contributed by assisting with the cognitive aspect of this project by conducting the initial content review and final review of the manuscript. PD contributed by providing the environment in which this research was conducted, assisted with the proposal and review of the manuscript. DW participated in the writing of the proposal, assisted with the interpretation of the analyses and assisted with the final review of the manuscript.

## Pre-publication history

The pre-publication history for this paper can be accessed here:


